# A Concise Synthesis of (−)‐Ambrox

**DOI:** 10.1002/open.202400006

**Published:** 2024-05-22

**Authors:** Bingyang Wang, Yanhui Liu, Chenyang Jia, Zhenfang Lan, Xuepeng Yang

**Affiliations:** ^1^ Collaborative Innovation Center for Food Production and Safety Zhengzhou University of Light Industry Zhengzhou University of Light Industry Dongfeng Road 5 Zhengzhou Henan 450002 People's Republic of China

**Keywords:** (−)-ambrox, synthesis, (*R*)-carvone

## Abstract

(−)‐Ambrox, a highly prized and commercially significant component of ambergris, finds widespread application in perfumery, cigarettes, cosmetics, and the food industry. Despite considerable attention to this research area over the years, an environmentally friendly and practical method for synthesizing (−)‐ambrox has remained elusive. This study presents a succinct and efficient approach to (−)‐ambrox synthesis, involving two consecutive alkylations at C‐6, followed by an acid‐catalyzed cyclization to give bicyclic ketones starting from (*R*)‐carvone. Subsequent reduction, Barton Vinyl Iodide synthesis, alkylation, and an acid‐catalyzed cyclization collectively achieved the synthesis of (−)‐ambrox with a satisfactory yield of 26.2 %.

## Graphical abstract

(−)‐Ambrox, a highly prized component of ambergris, finds widespread application in perfumery industry. This study presents an efficient approach to (−)‐ambrox synthesis, involving two consecutive alkylations followed by an acid‐catalyzed cyclization to give bicyclic ketones starting from (*R*)‐carvone. Subsequent reduction, Barton Vinyl Iodide synthesis, alkylation, and an acid‐catalyzed cyclization achieved the synthesis of (−)‐ambrox with a satisfactory yield of 26.2 %.

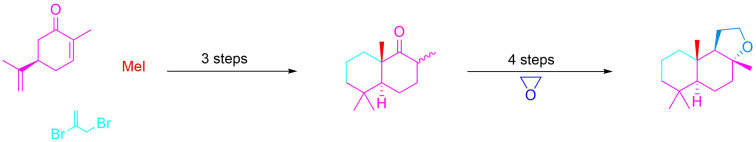

## Introduction

Ambergris, akin to civet, musk, and castoreum,^[1]^ stands out as one of the most valuable animal perfumes. Prior to its pivotal role in perfumery, ambergris garnered acclaim for its diverse biological activities, including body nourishment, use in aphrodisiacal wine blends, and medicinal applications to treat ailments such as headaches, colds, epilepsy, inflammation, and plague. In East Asia, it was even employed as a condiment. The substantial market demand for ambergris substitutes has drawn attention since ancient times. In the 1930s, Firmenich, the world's largest privately owned fragrance and flavor industry company, embarked on extensive research to identify the composition of ambergris and develop synthetic routes for producing the corresponding compounds.[^2^, ^3^, ^4^] Subsequently, synthetic (−)‐ambrox serves as an outstanding substitute for natural ambergris, finding widespread applications in perfumery, cigarettes, cosmetics, and the food industry.[^5^, ^6^, ^7^]

In perfumery, (−)‐ambrox is acknowledged as the archetype of all ambergris fragrances, typically employed as the base note of perfume compositions, with an extensive list of commercial products featuring this compound.[^4^, ^8^, ^9^, ^10^, ^11^, ^12^] As one of the most valuable ingredients in perfumery, the annual consumption of (−)‐ambrox exceeds 100 tons. Within the tobacco industry, (−)‐ambrox acts as an effective flavor enhancement and correction agent, imparting sweet, floral, and woody notes to cigarette vapor. In the cosmetics sector, (−)‐ambrox is widely utilized as a fragrance for hair, skin, and fabric and is known for its non‐irritating and non‐allergenic properties. In the realm of food chemistry, (−)‐ambrox holds the designation of a safe spice, as affirmed by the America Food Flavor and Extract Manufacturers Association (FEMA) and the United Nations Joint Expert Committee on Food Additives (JECFA).

**Figure 1:**
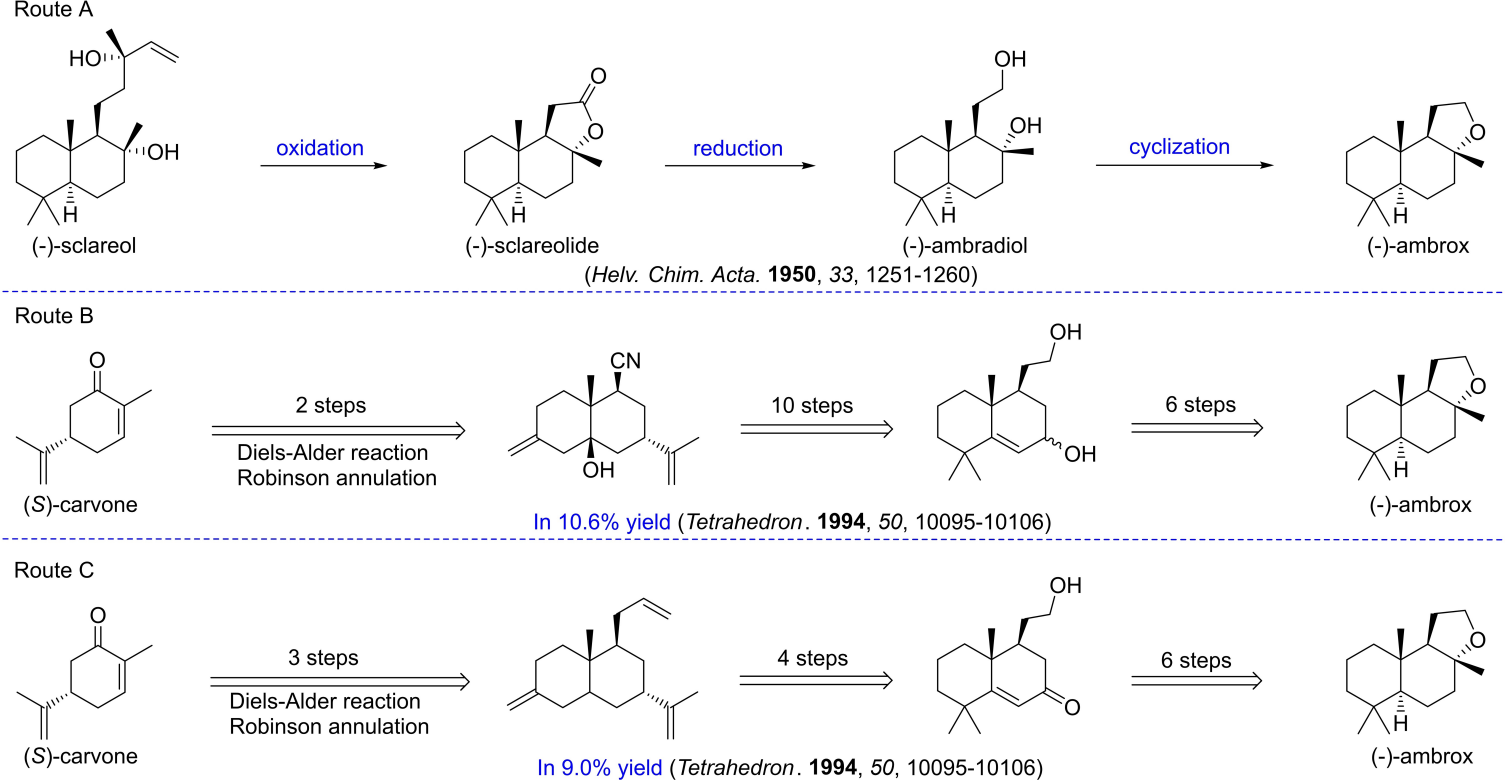
Approaches to (−)‐ambrox synthesis.

Due to the significant demand and high value associated with (−)‐ambrox, substantial attention has been devoted to this hot research topic over the past decades.[^13^, ^14^, ^15^, ^16^, ^17^, ^18^] Various linear and predominantly achiral polyene precursors, such as linalool,^[19]^ pseudoionone,[^20^, ^21^] (*E*)‐geranylacetone,^[22]^ (*E*, *E*)‐farnesol,[^23^, ^24^] and (*E*)‐*β*‐farnesene^[10]^ have been employed for (−)‐ambrox synthesis through polyene series cyclization. Semi‐synthesis primarily relies on natural monocyclic and polycyclic terpenes, including sclareol, *S*‐carvone^[25]^ (Figure 1), dysongensin A,^[26]^ oleanolic acid,^[9]^ labdanolic acid,[^27^, ^28^, ^29^] thujone,^[30]^ communic acid,^[31]^ (+)‐zabienol,^[32]^ levopimaric acid,^[33]^ (−)‐drimenol,^[34]^ *L*‐abietic acid,^[35]^ (+)‐nerolidol,^[36]^ *β*‐ionone,^[37]^ and manoyl oxide,^[38]^ as well as labdane type diterpenoids.[^39^, ^40^] However, most terpenes, except for sclareol as a starting material, exhibit drawbacks such as high processing costs, low yield, harsh reaction conditions (e. g., high pressure and temperature), or limited availability of starting materials, hindering their industrial realization and confining them primarily to academic research.

For commercial syntheses, (−)‐sclareol has been extensively employed in the production of (−)‐ambrox, benefitting from its commercially viable extraction from clary sage (Figure 1). The synthesis comprises three main stages: the oxidation of the side chain of sclareol to sclareolide, subsequent reduction to ambradiol, and the final acid‐catalyzed cyclization of the resulting diol to yield (−)‐ambrox.^[4]^ Despite the successful industrial production of (−)‐ambrox utilizing (−)‐sclareol as a starting material, these chemical routes encounter several challenges in terms of green chemistry. Presently, an environmentally friendly and practical preparation of (−)‐ambrox remains elusive.

(*R*)‐Carvone, a monocyclic terpene primarily derived from *Mentha spicata*, stands out as an economically feasible and easily accessible compound in the industrial domain. Obtainable in both enantiomerically pure forms, it serves as a cost‐effective and attractive chiron for the asymmetric total synthesis of diverse terpenes and related natural products. In 1989, Gesson^[41]^ documented the annulation of carvone, resulting in trans‐ and cis‐fused bicyclic synthons. Inspired by Gesson, we suggest that (*R*)‐carvone can be used as a cheap and readily available chiral starting material for asymmetric synthesis of (−)‐ambrox.

**Scheme 1:**
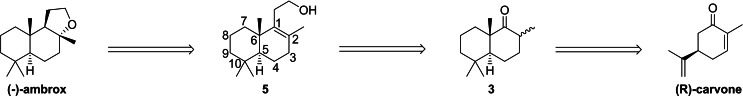
Retrosynthesis of (−)‐ambrox.

Our retrosynthetic plan to (−)‐ambrox starting from (*R*)‐carvone is shown in scheme 1. Two consecutive alkylations at C‐6 of (*R*)‐carvone, followed by an acid‐catalyzed cyclization and reduction, produce bicyclohexane skeleton **3** with the chiral centers at C‐6 in the correct configuration for the preparation of (−)‐ambrox. Compound **5** can be approached through Barton Vinyl Iodide synthesis and alkylation from **3**. (−)‐Ambrox can be synthesized from compound **5** through an acid‐catalyzed cyclization.

**Scheme 2:**
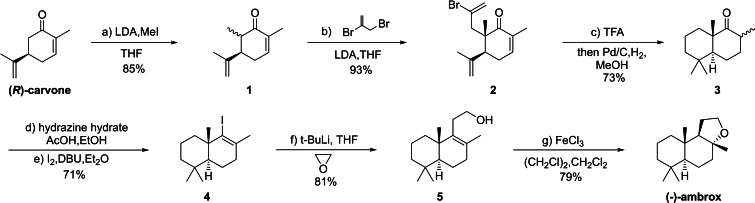
Synthesis of (−)‐ambrox. Reagents and conditions: a) LDA (1.4 equiv.), MeI (3.5 equiv.), THF, −25 °C to RT, 12 hours, 85 %; b) LDA (1.5 equiv.), 2,3‐dibromopropene (2.0 equiv.), THF, −25 °C to RT, 12 hours, 93 %; c) TFA, 3 days; then Pd/C, H_2_, MeOH, 4 days, 73 %; d) hydrazine hydrate (10.0 equiv.), AcOH (5.0 equiv.), EtOH, 80 °C, reflux, 16 hours; e) DBU (20.0 equiv.), I_2_ (2.2 equiv.), ether, 25 °C, 30 minutes, 71 % for two steps; f) *t*‐BuLi (1.4 equiv.), ethylene oxide (3.6 equiv.), THF, −78 °C to 0 °C, 3 hours, 81 %; g) FeCl_3_ (0.5 equiv.), DCM, (CH_2_Cl)_2_, 23 °C, 2 hours, 79 %. THF=Tetrahydrofuran, DCM=Dichloromethane, LDA=Lithium diisopropylamide, TFA=Trifluoroacetic acid.

Building upon Gesson,^[41]^ we selected (*R*)‐carvone as the starting material for the enantioselective synthesis of (−)‐ambrox (Scheme 2). The construction of the bicyclohexane ring, bearing a gem‐dimethyl group characteristic of (−)‐ambrox, involved two consecutive alkylations at C‐6 of (*R*)‐carvone, followed by an acid‐catalyzed cyclization. The kinetic enolate of (*R*)‐carvone was prepared and methylated under LDA‐THF conditions, yielding compound **1** in an 85 % yield, which could be converted to the expected *α* enolate under the same conditions. We also tried other bases such as triethylamine, potassium carbonate, NaH or butyl lithium, with on reaction or a dimethyl substitution as the major product. The ensuing enolate reacted diastereoselectively with 2‐bromo‐allylic bromide, providing compound **2** in a 93 % yield. The steric hindrance imposed by the isopropenyl group on one side of the dienolate determined the anticipated stereochemistry of the newly formed quaternary carbon.

The carbocation cyclization catalyzed by Lewis acid, as reported by Lansbury,^[42]^ was achieved by stirring compound **2** in trifluoroacetic acid at room temperature for 3 days, successfully generating the bicyclohexane skeleton. The stereoselectivity of the second alkylation step controlled the stereochemistry of the newly formed ring junction. Subsequent reduction with Pd/C under H_2_ atmosphere at normal pressure was carried out after removing trifluoroacetic acid under reduced pressure, successfully reducing the halogen and the two carbon‐carbon double bonds.

Addition of vinylmagnesium bromide to the ketone moiety in compound **3** gave a diastereomeric mixture of alcohol which was dehydrated with thionyl chloride to diene. The subsequent hydroboration reduction with diisoamylborane, 9‐borabicyclo [3.3.1] nonane or borane‐THF complex of the terminal olefin provided no desired product or only a small amount of the primary alcohol **5**. While the use of Wittig reagent such as Ph_3_(Me)PI to elongate the carbon chain provided only a low yield product perhaps due to the relatively large steric resistance near the carbonyl group. Finally, the hydrazone of the ketone **3** was formed by stirring compound **3** and hydrazine hydrate in ethanol for 16 hours. The elimination of nitrogen gas was achieved through treatment with iodine and 1,8‐diazabicyclo [5.4.0] undec‐7‐ene (DBU) in ether, yielding the vinyl iodide **4** in 71 % yield. Treating vinyl iodide **4** with *t*‐BuLi through lithiation, followed by reaction with ethylene oxide, produced the primary alcohol **5** in 81 % yield. The final cyclization to (−)‐ambrox was extensively investigated using FeCl_3_ or p‐toluenesulfonic acid and nitromethane.^[11]^ Catalyzed by FeCl_3_, (−)‐ambrox was obtained in a 79 % yield.

## Conclusions

We have outlined a concise and enantioselective seven‐step route to access (−)‐ambrox from the readily available and cost‐effective (*R*)‐carvone. This synthesis involves two consecutive alkylations, an acid‐catalyzed cyclization, reduction, and Barton Vinyl Iodide Synthesis followed by alkylation and another acid‐catalyzed cyclization, ultimately yielding (−)‐ambrox in a 26.2 % yield. This is the first time that (*R*)‐carvone was used as starting material for the synthesis of (−)‐ambrox. Compared with Various linear, predominantly achiral polyene precursors or some terpenoids, the synthesis route was concise with high atomic economy and a satisfactory yield. The stereochemistry of (−)‐ambrox was well controlled. Compared with (−) ‐sclareol as starting material, some oxidants that pollute the environment were avoided, but the use of tert‐butyl lithium limited large‐scale preparation of (−)‐ambrox. We are still doing research in our lab in order to further simplify the preparation of (−)‐ambrox.

## Experimental Section

All reactions involving air or moisture sensitive reagents or intermediates were carried out under an argon atmosphere with dry solvents under anhydrous conditions, unless otherwise noted. Reagents were purchased at the highest commercial quality and used without further purification, unless otherwise stated. Solvents purification was conducted according to Purification of Laboratory Chemicals (Peerrin, D. D.; Armarego, W. L. and Perrins, D. R., Pergamon Press: Oxford, 1980). Tetrahydrofuran was distilled from sodium/benzophenone. Dichloromethane was distilled from calcium hydride. Yields refer to isolated compounds unless otherwise stated NMR spectra were recorded on a Brüker AVANCE 600 (^1^H: 600 MHz, ^13^C: 151 MHz) instrument. Chemical shifts were reported in parts per million (ppm) with respect to the residual solvent signal CDCl_3_ (^1^H NMR: δ=7.26; ^13^C NMR: δ=77.00). Peak multiplicities were reported as follows: s=singlet, d=doublet, t=triplet, q=quartet, dd=doublet of doublets, td=triplet of doublets, dt=doublet of triplets, ddd=doublet of doublet of doublets, m=multiplet, br=broad signal. High resolution mass spectra (HRMS) were recorded on an Agilent Mass spectrometer using ESI‐TOF (electrospray ionization‐time of flight).

**Procedure for the synthesis of compound 1**. To a stirred solution of (*R*)‐carvone (10.0 g, 66.6 mmol, 1.0 equiv.) in anhydrous THF (340.0 mL) at −25 °C was slowly added LDA (46.6 mL, 2 mol/L in THF, 1.4 equiv.). The resulting solution was stirred at −25 °C for 2 hours. Then it was added MeI (33.1 g, 233.0 mmol, 3.5 equiv.) and stirred for 1 hour at −25 °C before the cooling bath was removed. After being stirred at room temperature for 12 hours, a saturated solution of NH_4_Cl (250 mL) was added. The mixture was extracted with EtOAc (3×300 mL). The combined organic extracts were washed with saturated brine, and dried over anhydrous Na_2_SO_4_. The solvent was removed under vacuum, and residue was purified using column chromatography on silica gel (petroleum ether: EtOAc=50 : 1 v/v) to give compound **1** (9.3 g, 56.6 mmol, 85 %).^1^H NMR (600 MHz, Chloroform‐d): δ 6.75–6.66 (m, 1H), 4.91 (d, *J*=1.4 Hz, 1H), 4.73 (d, *J*=1.3 Hz, 1H), 2.73–2.65 (m, 2H), 2.48–2.43 (m, 1H), 2.33–2.26 (m, 1H), 1.80–1.76 (m, 3H), 1.70 (d, *J*=1.4 Hz, 3H), 0.92 (d, *J*=7.3 Hz, 3H); ^13^C NMR (151 MHz, Chloroform‐d): δ 203.57, 144.93, 144.03, 133.65, 111.46, 44.73, 42.96, 26.25, 21.89, 15.98, 10.47; HRMS (APCI): calculated for C_11_H_17_O [M+H]^+^ 165.1274, found 165.1277.

**Procedure for the synthesis of compound 2**. To a stirred solution of **1** (8.0 g, 48.7 mmol, 1.0 equiv.) in anhydrous THF (250.0 mL) at −25 °C was slowly added LDA (36.0 mL, 2 mol/L in THF, 1.5 equiv.). The resulting solution was stirred at −25 °C for 2 hours, then it was added 2,3‐dibromopropene (19.5 g, 97.4 mmol, 2.0 equiv.) and stirred at −25 °C for 1 hour before the cooling bath was removed. After being stirred at room temperature for 12 hours, a saturated solution of NH_4_Cl (200 mL) was added. The mixture was extracted with EtOAc (3 x 300 mL). The combined organic extracts were washed with saturated brine, and dried over anhydrous Na_2_SO_4_. The solvent was removed under vacuum, and residue was purified using column chromatography on silica gel (petroleum ether: EtOAc=50 : 1 v/v) to give compound **2** (12.8 g, 45.3 mmol, 93 %).^1^H NMR (600 MHz, Chloroform‐d): δ 6.63–6.55 (m, 1H), 5.57 (d, *J*=1.9 Hz, 1H), 5.47 (dt, *J*=2.0, 1.0 Hz, 1H), 4.82–4.67 (m, 2H), 2.90 (dd, *J*=6.2, 4.3 Hz, 1H), 2.84 (dd, *J*=15.0, 1.1 Hz, 1H), 2.75–2.68 (m, 2H), 2.30 (dtd, *J*=19.9, 4.4, 2.1 Hz, 1H), 1.79 (q, *J*=1.9 Hz, 3H), 1.60 (s, 3H), 1.09 (s, 3H). ^13^C NMR (151 MHz, Chloroform‐d): δ 202.61, 145.81, 141.38, 134.51, 128.45, 121.14, 114.45, 49.34, 48.83, 48.09, 28.47, 21.98, 19.54, 16.52. HRMS (APCI): calculated for C_14_H_20_BrO [M+H]^+^ 283.0692, found 283.0685.

**Procedure for the synthesis of compound 3**. To a stirred solution of **2** (10.0 g, 35.3 mmol, 1.0 equiv.) at room temperature was added TFA (trifluoroacetic acid, 35.0 mL). After being stirred at room temperature for 3 days, TFA was removed under reduced pressure, the residue was dissolved in 50.0 mL MeOH, Pd/C (10 %, 1.6 g, 1.5 mmol) was added. Then replaced it three times with H_2_. After the solution was stirred at room temperature for 4 days, filtered and concentrated under vacuum. The residue was purified using column chromatography on silica gel (petroleum ether: EtOAc=100 : 1 v/v) to give compound **3** (5.7 g, 25.8 mmol, 73 %).^1^H NMR (600 MHz, Chloroform‐d): δ 2.70–2.63 (m, 1H), 2.12–2.04 (m, 1H), 1.77–1.64 (m, 2H), 1.54 (td, *J*=11.7, 9.5, 4.5 Hz, 4H), 1.40‐1.34 (m, 1H), 1.20 (td, *J*=12.9, 5.3 Hz, 1H), 1.16–1.12 (m, 1H), 1.12 (s, 3H), 1.09 (dd, *J*=11.5, 4.1 Hz, 1H), 0.96 (d, *J*=6.5 Hz, 3H), 0.91 (s, 3H), 0.86 (s, 3H). ^13^C NMR (151 MHz, Chloroform‐d): δ 216.80, 54.19, 48.78, 41.57, 39.89, 35.72, 34.19, 33.20, 33.04, 22.01, 21.32, 18.75, 18.15, 15.02. HRMS (APCI): calculated for C_14_H_25_O [M+H]^+^ 209.1900, found 209.1904.

**Procedure for the synthesis of compound 4**. To a stirred solution of **3** (4.0 g, 24.0 mmol, 1.0 equiv.), hydrazine hydrate (85 %, 9.1 g, 240.0 mmol, 10.0 equiv.), acetic acid (7.2 g, 120.0 mmol, 5.0 equiv.), and ethyl alcohol (120.0 mL) was refluxed for 21 hours. Then a saturated solution of NaHCO_3_ (100 mL) was added. The mixture was extracted with EtOAc (3 x 150 mL). The combined organic extracts were washed with saturated brine, and dried over anhydrous Na_2_SO_4_. The solvent was removed under vacuum to furnish the hydrazone. To a stirred solution of hydrazone and 1,8‐diazabicyclo [5.4.0] undec‐7‐ene (DBU, 73.1 g, 480.0 mmol, 20.0 equiv.) in ether (6.0 mL) at 23 °C was added iodine (13.4 g, 52.6 mmol, 2.2 equiv.). The mixture was stirred at 23 °C for 30 minutes, then a saturated solution of NaHCO_3_ (100 mL) was added and washed with 10 % Na_2_S_2_O_3_ (100 mL). The mixture was extracted with EtOAc (3×300 mL). The combined organic extracts were washed with saturated brine, and dried over anhydrous Na_2_SO_4_. The solvent was removed under vacuum, and residue was purified using column chromatography on silica gel (petroleum) to give compound **4** (5.4 g, 17.0 mmol, 71 %).^1^H NMR (600 MHz, Chloroform‐d): δ 2.31–2.17 (m, 2H), 1.84 (s, 3H), 1.71–1.66 (m, 1H), 1.54 (d, 2H), 1.52–1.42 (m, 2H), 1.42–1.36 (m, 1H), 1.34 (dd, *J*=12.6, 1.9 Hz, 1H), 1.15 (td, *J*=13.4, 4.5 Hz, 1H), 1.08 (td, *J*=13.0, 4.2 Hz, 1H), 1.00 (s, 3H), 0.92 (s, 3H), 0.84 (s, 3H).^13^C NMR (151 MHz, Chloroform‐d): δ 136.42, 121.99, 52.64, 44.50, 42.50, 41.75, 35.34, 33.81, 32.88, 30.72, 21.43, 19.78, 19.53, 18.88. HRMS (EI): calculated for C_14_H_24_I [M+H]^+^ 318.0839, found 318.0842.

**Procedure for the synthesis of compound 5**. To a stirred solution of vinyl iodide **4** (4.0 g, 12.6 mmol, 1.0 equiv.) in dry THF (60.0 mL) at −78 °C was slowly added *t*‐BuLi (29.0 mL, 1.3 mol/L in pentane, 1.4 equiv.). The resulting mixture was stirred at −78 °C for 1.5 hours, then it was added ethylene oxide (15.1 mL, 3.0 mol/L in THF, 45.3 mmol, 3.6 equiv.) and stirred at −78 °C for 1 hour. Then the solution was slowly warmed to 0 °C for 3 hours, a saturated solution of NH_4_Cl (60 mL) was added. The mixture was extracted with EtOAc (3×300 mL). The combined organic extracts were washed with saturated brine, and dried over anhydrous Na_2_SO_4_. The solvent was removed under vacuum, and residue was purified using column chromatography on silica gel (petroleum ether: EtOAc=10: 1 v/v) to give compound **5** (2.4 g, 10.2 mmol, 81 %). ^1^H NMR (600 MHz, Chloroform‐d): δ 3.66–3.53 (m, 2H), 2.45–2.33 (m, 1H), 2.24 (ddd, *J*=13.4, 9.9, 5.7 Hz, 1H), 2.10–1.92 (m, 2H), 1.87–1.81 (m, 1H), 1.67–1.56 (m, 5H), 1.50–1.38 (m, 4H), 1.17–1.04 (m, 3H), 0.95 (s, 3H), 0.88 (s, 3H), 0.83 (s, 3H). ^13^C NMR (151 MHz, Chloroform‐d): δ 136.17, 128.55, 62.63, 51.66, 41.70, 38.66, 37.14, 33.64, 33.31, 33,29, 31.46, 21.67, 20.06, 19.91, 19.00, 18.98. HRMS (APCI): calculated for C_16_H_29_O [M+H]^+^ 237.2213, found 237.2219.

**Procedure for the synthesis of (−)‐ambrox**. To a stirred solution of **5** (2.0 g, 8.5 mmol, 1.0 equiv.) in 1,2‐dichloroethane (16.0 mL) and CH_2_Cl_2_ (8.0 mL) was added SiO_2_ (70–220 μm) (507.6 mg, 8.5 mmol, 1.0 equiv.) at 23 °C. Then FeCl_3_ (686.1 mg, 4.2 mmol, 0.5 equiv.) was added. After being stirred for 20 minutes, a saturated solution of 10 % HCl (30 mL) was added. The mixture was extracted with EtOAc (3×30 mL). The combined organic extracts were washed with water (30 mL), NaHCO_3_ (2×30 mL) and saturated brine, and dried over anhydrous Na_2_SO_4_. The solvent was removed under vacuum, and residue was purified using column chromatography on silica gel (petroleum ether: EtOAc=30 : 1 v/v) to give (−)‐ambrox (1.6 g, 6.7 mmol, 79 %).

^1^H NMR (600 MHz, Chloroform‐d): δ 3.66–3.53 (m, 2H), 2.45–2.33 (m, 1H), 2.24 (ddd, *J*=13.4, 9.9, 5.7 Hz, 1H), 2.10–1.92 (m, 2H), 1.87–1.81 (m, 1H), 1.67–1.56 (m, 5H), 1.50–1.38 (m, 4H), 1.17–1.04 (m, 3H), 0.95 (s, 3H), 0.88 (s, 3H), 0.83 (s, 3H). ^13^C NMR (151 MHz, Chloroform‐d): δ 79.93, 64.98, 60.11, 57.25, 42.43, 39.95, 39.74, 36.19, 33.58, 33.07, 22.63, 21.13, 21.13, 20.65, 18.40, 15.04. HRMS (APCI): calculated for C_16_H_29_O [M+H]^+^ 237.2213, found 237.2218.

## Supporting Information

The authors have cited additional references within the Supporting Information.

## Conflict of Interests

The authors declare no conflict of interest.

1

## Supporting information

As a service to our authors and readers, this journal provides supporting information supplied by the authors. Such materials are peer reviewed and may be re‐organized for online delivery, but are not copy‐edited or typeset. Technical support issues arising from supporting information (other than missing files) should be addressed to the authors.

Supporting Information

## Data Availability

The data that support the findings of this study are available in the supplementary material of this article.
