# Supplementary material for: A Concise Synthesis of (−)‐Ambrox
Source: ChemistryOpen. 2024 May 22;13(9):e202400006. doi: 10.1002/open.202400006 (PMC11467728; doi:10.1002/open.202400006)
Supplement: Supplementary file 1 — Supporting Information [file OPEN-13-e202400006-s001.pdf]

ChemistryOpen

Supporting Information

A Concise Synthesis of (−)-Ambrox

Bingyang Wang, Yanhui Liu, Chenyang Jia, Zhenfang Lan, and Xuepeng Yang*

ChemistryOpen

Supporting Information

A concise synthesis of (-)-ambrox

Bingyang Wang, Yanhui Liu, Chenyang Jia, Zhenfang Lan, Xuepeng Yang,*

Content:

Experimental Procedures 1

NMR Spectra of All Compounds..6

Experimental Procedures

All reactions involving air or moisture sensitive reagents or intermediates were carried out under an argon atmosphere with dry solvents under anhydrous conditions, unless otherwise noted. Reagents were purchased at the highest commercial quality and used without further purification, unless otherwise stated. Solvents purification was conducted according to Purification of Laboratory Chemicals (Peerrin, D.D.; Armarego, W. L. and Perrins, D. R., Pergamon Press: Oxford, 1980). Tetrahydrofuran was distilled from sodium/benzophenone. Dichloromethane was distilled from calcium hydride. Yields refer to isolated compounds unless otherwise stated. NMR spectra were recorded on a Brüker AVANCE 600 (^{1}H: 600 MHz, ^{13}C: 151MHz) instrument. Chemical shifts were reported in parts per million (ppm) with respect to the residual solvent signal $CDCl_3$ (^{1}H NMR: δ = 7.26; ^{13}C NMR: δ = 77.00). Peak multiplicities were reported as follows: s = singlet, d = doublet, t = triplet, q = quartet, dd = doublet of doublets, td = triplet of doublets, dt = doublet of triplets, ddd = doublet of doublet of doublets, m = multiplet, br = broad signal. High resolution mass spectra (HRMS) were recorded on an Agilent Mass spectrometer using ESI-TOF (electrospray ionization-time of flight).

Procedure for the synthesis of compound 1. To a stirred solution of (*R*)-carvone (10.0 g, 66.6 mmol, 1.0 equiv.) in anhydrous THF (340.0 mL) at -25 °C was slowly added LDA (46.6 mL, 2 mol/L in THF, 1.4 equiv.). The resulting solution was stirred at -25 °C for 2 hours. Then it was added MeI (33.1 g, 233.0 mmol, 3.5 equiv.) and stirred for 1 hour at -25 °C before the cooling bath was removed. After being stirred at room temperature for 12 hours, a saturated solution of NH_4Cl (250 mL) was added. The mixture was extracted with EtOAc (3 x 300 mL). The combined organic extracts were washed with saturated brine, and dried over anhydrous Na_2SO_4. The solvent was removed under vacuum,

and residue was purified using column chromatography on silica gel (petroleum ether: EtOAc = 50:1 v/v) to give compound **1** (9.3 g, 56.6 mmol, 85%).

^{1}H NMR (600 MHz, Chloroform-*d*): δ 6.75 – 6.66 (m, 1H), 4.91 (d, *J* = 1.4 Hz, 1H), 4.73 (d, *J* = 1.3 Hz, 1H), 2.73 – 2.65 (m, 2H), 2.48 – 2.43 (m, 1H), 2.33 – 2.26 (m, 1H), 1.80 – 1.76 (m, 3H), 1.70 (d, *J* = 1.4 Hz, 3H), 0.92 (d, *J* = 7.3 Hz, 3H).

^{13}C NMR (151 MHz, Chloroform-d): δ 203.57, 144.93, 144.03, 133.65, 111.46, 44.73, 42.96, 26.25, 21.89, 15.98, 10.47;

HRMS (APCI): calculated for $C_{11}H_{17}O$ $[M+H]^+$ 165.1274, found 165.1277.

Procedure for the synthesis of compound 2. To a stirred solution of **1** (8.0 g, 48.7 mmol, 1.0 equiv.) in anhydrous THF (250.0 mL) at -25 °C was slowly added LDA (36.0 mL, 2 mol/L in THF, 1.5 equiv.). The resulting solution was stirred at -25 °C for 2 hours, then it was added 2,3-dibromopropene (19.5 g, 97.4 mmol, 2.0 equiv.) and stirred at -25 °C for 1 hour before the cooling bath was removed. After being stirred at room temperature for 12 hours, a saturated solution of NH_4Cl (200 mL) was added. The mixture was extracted with EtOAc (3 x 300 mL). The combined organic extracts were washed with saturated brine, and dried over anhydrous Na_2SO_4. The solvent was removed under vacuum, and residue was purified using column chromatography on silica gel (petroleum ether: EtOAc = 50:1 v/v) to give compound **2** (12.8 g, 45.3 mmol, 93%).

^{1}H NMR (600 MHz, Chloroform-*d*): δ 6.63 – 6.55 (m, 1H), 5.57 (d, *J* = 1.9 Hz, 1H), 5.47 (dt, *J* = 2.0, 1.0 Hz, 1H), 4.82 – 4.67 (m, 2H), 2.90 (dd, *J* = 6.2, 4.3 Hz, 1H), 2.84 (dd, *J* = 15.0, 1.1 Hz, 1H), 2.75 – 2.68 (m, 2H), 2.30 (dtd, *J* = 19.9, 4.4, 2.1 Hz, 1H), 1.79 (q, *J* = 1.9 Hz, 3H), 1.60 (s, 3H), 1.09 (s, 3H).

^{13}C NMR (151 MHz, Chloroform-d): δ 202.61, 145.81, 141.38, 134.51, 128.45, 121.14, 114.45, 49.34, 48.83, 48.09, 28.47, 21.98, 19.54, 16.52.

HRMS (APCI): calculated for $C_{14}H_{20}BrO$ $[M+H]^+$ 283.0692, found 283.0685.

Procedure for the synthesis of compound 3. To a stirred solution of **2** (10.0 g, 35.3 mmol, 1.0 equiv.) at room temperature was added TFA (trifluoroacetic acid, 35.0 mL). After being stirred at room temperature for 3 days, TFA was removed under reduced pressure, the residue was dissolved in 50.0 mL MeOH, Pd/C (10%, 1.6 g, 1.5 mmol) was added. Then replaced it three times with H_2. After the solution was stirred at room temperature for 4 days, filtered and concentrated under vacuum. The residue was purified using column chromatography on silica gel (petroleum ether: EtOAc = 100:1 v/v) to give compound **3** (5.7 g, 25.8 mmol, 73%).

^{1}H NMR (600 MHz, Chloroform-*d*): δ 2.70 – 2.63 (m, 1H), 2.12 – 2.04 (m, 1H), 1.77 – 1.64 (m, 2H), 1.56 – 1.51 (m, 4H), 1.40-1.34 (m, 1H), 1.20 (td, *J* = 12.9, 5.3 Hz, 1H), 1.16 – 1.12 (m, 1H), 1.12 (s, 3H), 1.09 (dd, *J* = 11.5, 4.1 Hz, 1H), 0.96 (d, *J* = 6.5 Hz, 3H), 0.91 (s, 3H), 0.86 (s, 3H).

^{13}C NMR (151 MHz, Chloroform-d): δ 216.80, 54.19, 48.78, 41.57, 39.89, 35.72, 34.19, 33.20, 33.04, 22.01, 21.32, 18.75, 18.15, 15.02.

HRMS (APCI): calculated for $C_{14}H_{25}O$ $[M+H]^+$ 209.1900, found 209.1904.

Procedure for the synthesis of compound 4. To a stirred solution of **3** (4.0 g, 24.0 mmol, 1.0 equiv.), hydrazine hydrate (85%, 9.1 g, 240.0 mmol, 10.0 equiv.), acetic acid (7.2 g, 120.0 mmol, 5.0 equiv.), and ethyl alcohol (120.0 mL) was refluxed for 21 hours. Then a saturated solution of $NaHCO_3$ (100 mL) was added. The mixture was extracted with EtOAc (3 x 150 mL). The combined organic extracts were washed with saturated brine, and dried over anhydrous Na_2SO_4. The solvent was removed under vacuum to furnish the hydrazone. To a stirred solution of hydrazone and 1,8-diazabicyclo [5.4.0] undec-7-ene (DBU, 73.1 g, 480.0 mmol, 20.0 equiv.) in ether (6.0 mL) at 23 °C was added iodine (13.4 g, 52.6 mmol, 2.2 equiv.). The mixture was stirred at 23 °C for 30 minutes, then a saturated solution of $NaHCO_3$ (100 mL) was added and washed with 10% $Na_2S_2O_3$ (100 mL). The mixture was extracted with EtOAc (3 x 300 mL). The combined organic extracts were washed with saturated brine, and dried over anhydrous Na_2SO_4. The solvent was removed under vacuum, and residue was

purified using column chromatography on silica gel (petroleum) to give compound **4** (5.4 g, 17.0 mmol, 71%).

^{1}H NMR (600 MHz, Chloroform-d): δ 2.31 – 2.17 (m, 2H), 1.84 (s, 3H), 1.71 – 1.66 (m, 1H), 1.54 (d, 2H), 1.52 – 1.42 (m, 2H), 1.42 – 1.36 (m, 1H), 1.34 (dd, *J* = 12.6, 1.9 Hz, 1H), 1.15 (td, *J* = 13.4, 4.5 Hz, 1H), 1.08 (td, *J* = 13.0, 4.2 Hz, 1H), 1.00 (s, 3H), 0.92 (s, 3H), 0.84 (s, 3H).

^{13}C NMR (151 MHz, Chloroform-d): δ 136.42, 121.99, 52.64, 44.50, 42.50, 41.75, 35.34, 33.81, 32.88, 30.72, 21.43, 19.78, 19.53, 18.88.

HRMS (EI): calculated for $C_{14}H_{24}I$ $[M+H]^+$ 318.0839, found 318.0842

Procedure for the synthesis of compound 5. To a stirred solution of vinyl iodide **4** (4.0 g, 12.6 mmol, 1.0 equiv.) in dry THF (60.0 mL) at -78 °C was slowly added *t*-BuLi (29.0 mL, 1.3 mol/L in pentane, 1.4 equiv.). The resulting mixture was stirred at -78 °C for 1.5 hours, then it was added ethylene oxide (15.1 mL, 3.0 mol/L in THF, 45.3 mmol, 3.6 equiv.) and stirred at -78 °C for 1 hour. Then the solution was slowly warmed to 0 °C for 3 hours, a saturated solution of NH_4Cl (60 mL) was added. The mixture was extracted with EtOAc (3 x 300 mL). The combined organic extracts were washed with saturated brine, and dried over anhydrous Na_2SO_4. The solvent was removed under vacuum, and residue was purified using column chromatography on silica gel (petroleum ether: EtOAc = 10: 1 v/v) to give compound **5** (2.4 g, 10.2 mmol, 81%).

^{1}H NMR (600 MHz, Chloroform-d): δ 3.66 – 3.53 (m, 2H), 2.45 – 2.33 (m, 1H), 2.24 (ddd, *J* = 13.4, 9.9, 5.7 Hz, 1H), 2.10 – 1.92 (m, 2H), 1.87 – 1.81 (m, 1H), 1.67 – 1.56 (m, 5H), 1.50 – 1.38 (m, 4H), 1.17 – 1.04 (m, 3H), 0.95 (s, 3H), 0.88 (s, 3H), 0.83 (s, 3H).

^{13}C NMR (151 MHz, Chloroform-d): δ 136.17, 128.55, 62.63, 51.66, 41.70, 38.66, 37.14, 33.64, 33.31, 33,29, 31.46, 21.67, 20.06, 19.91, 19.00, 18.98.

HRMS (APCI): calculated for $C_{16}H_{29}O$ $[M+H]^+$ 237.2213, found 237.2219.

Procedure for the synthesis of (-)-ambrox. To a stirred solution of **5** (2.0 g, 8.5 mmol, 1.0 equiv.) in 1,2-dichloroethane (16.0 mL) and CH_2Cl_2 (8.0 mL) was added SiO_2 (507.6 mg, 8.5 mmol, 1.0 equiv.) at 23 °C. Then $FeCl_3$ (686.1 mg, 4.2 mmol, 0.5 equiv.) was added. After being stirred for 20 minutes, a saturated solution of 10% HCl (30 mL) was added. The mixture was extracted with EtOAc (3 x 30 mL). The combined organic extracts were washed with water (30 mL), $NaHCO_3$ (2 x 30 mL) and saturated brine, and dried over anhydrous Na_2SO_4. The solvent was removed under vacuum, and residue was purified using column chromatography on silica gel (petroleum ether: EtOAc = 30:1 v/v) to give (-)-ambrox (1.6 g, 6.7 mmol, 79%). $[\alpha]^{28.6}_{D}$: -27 (c = 0.1, MeOH).

1H NMR (600 MHz, Chloroform-d): δ 3.91 (td, J = 8.5, 3.8 Hz, 1H), 3.82 (q, J = 8.2 Hz, 1H), 1.94 (dt, J = 11.6, 3.2 Hz, 1H), 1.78 – 1.62 (m, 4H), 1.49 – 1.37 (m, 5H), 1.33 – 1.15 (m, 2H), 1.08 (s, 3H), 1.06 – 0.93 (m, 2H), 0.87 (s, 3H), 0.83 (s, 3H), 0.83 (s, 3H).

^{13}C NMR (151 MHz, Chloroform-d): δ 79.93, 64.98, 60.11, 57.25, 42.43, 39.95, 39.74, 36.19, 33.58, 33.07, 22.63, 21.13, 21.13, 20.65, 18.40, 15.04.

HRMS (APCI): calculated for $C_{16}H_{29}O$ $[M+H]^+$ 237.2213, found 237.2218.

NMR Spectra of All Compounds

Compound 1 ^{1}H NMR

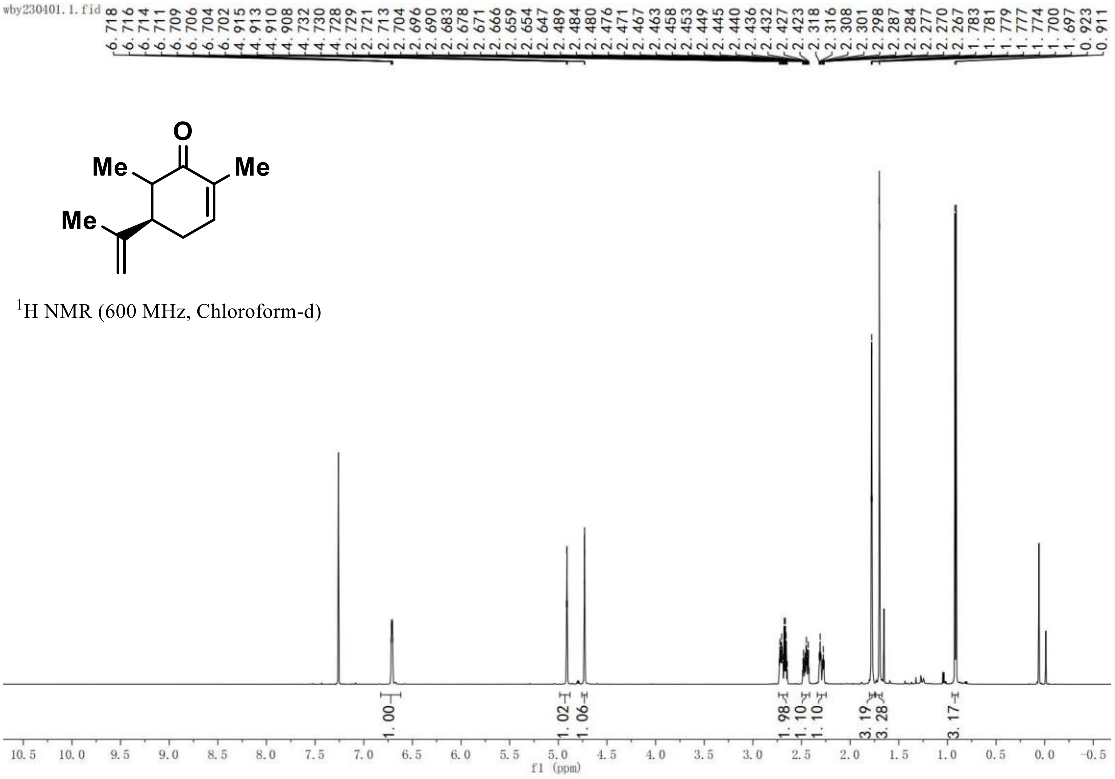

Compound 1 ^{13}C NMR

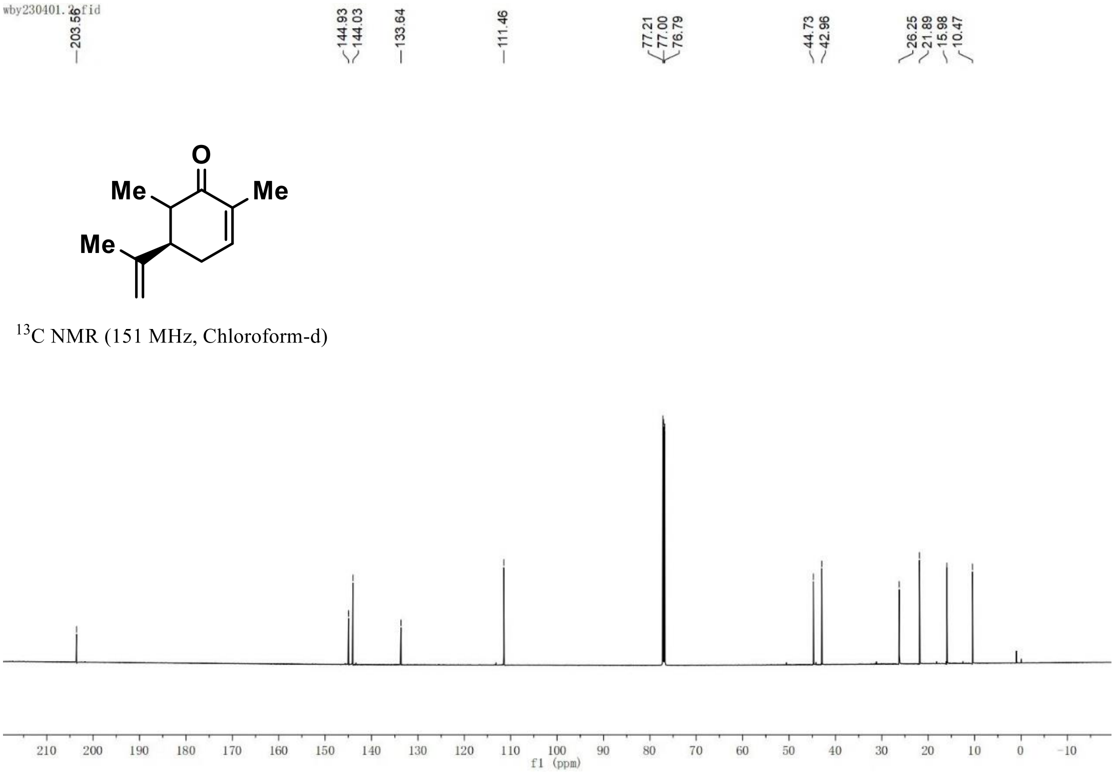

Compound 2 ^{1}H NMR

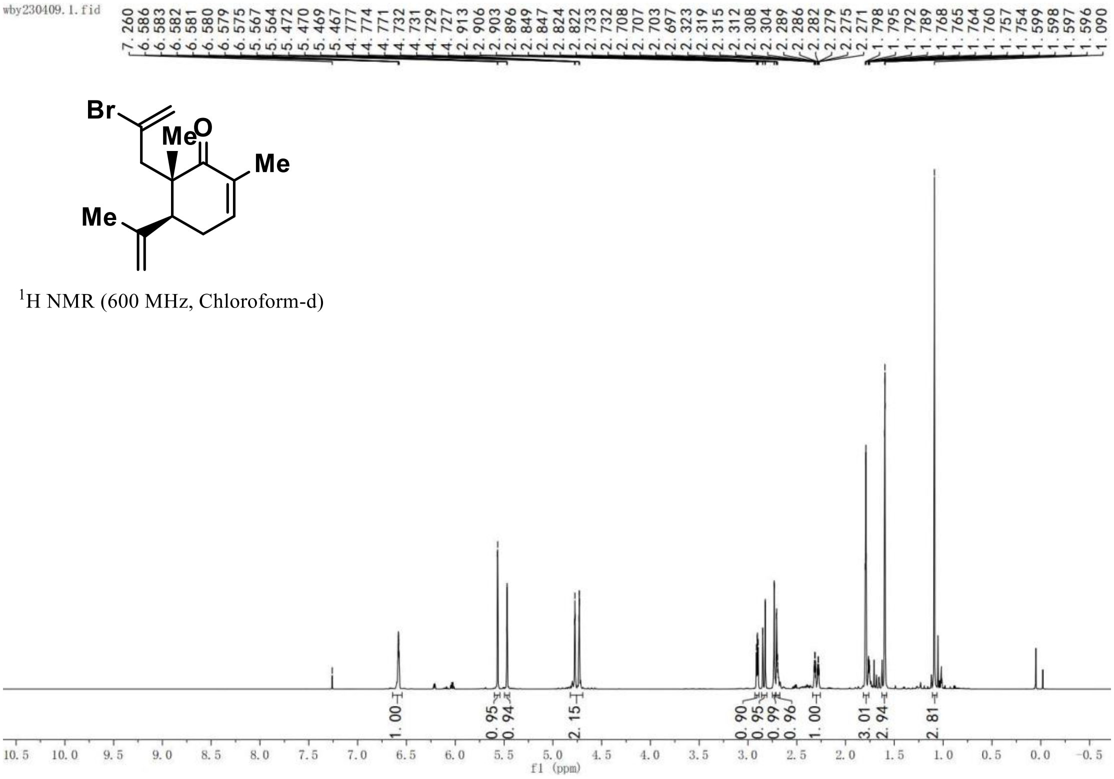

Compound 2 ^{13}C NMR

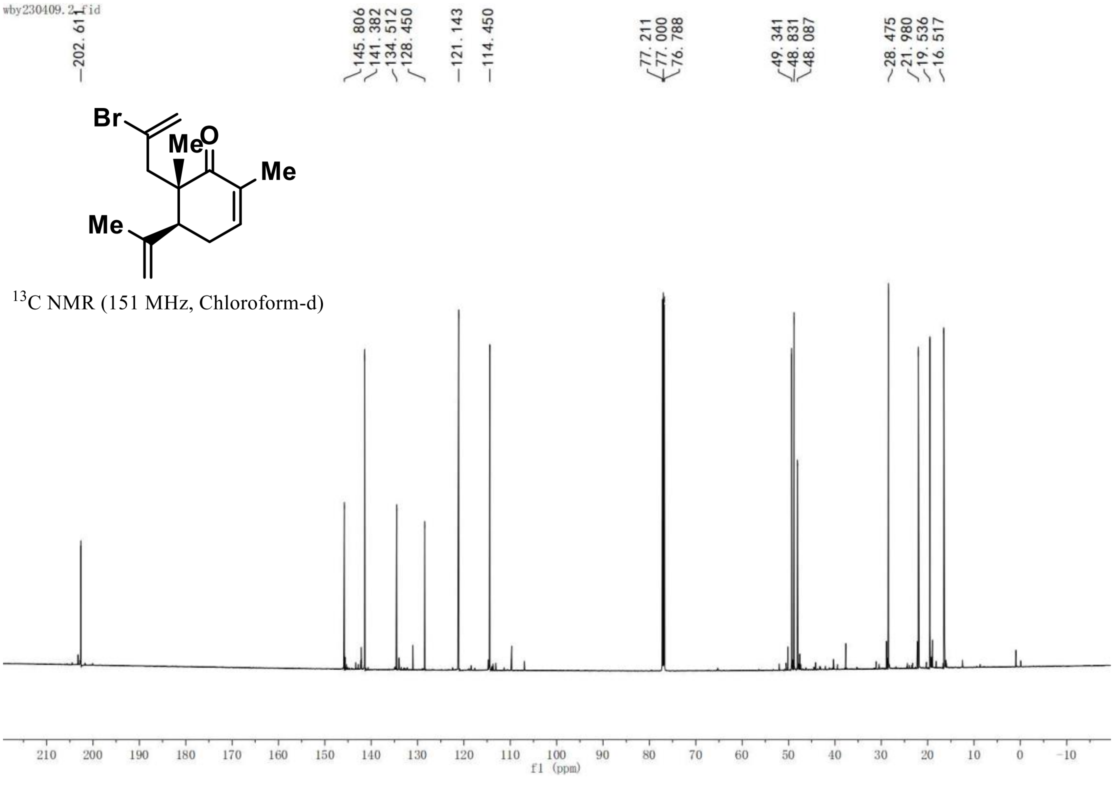

Compound 3 ^{1}H NMR

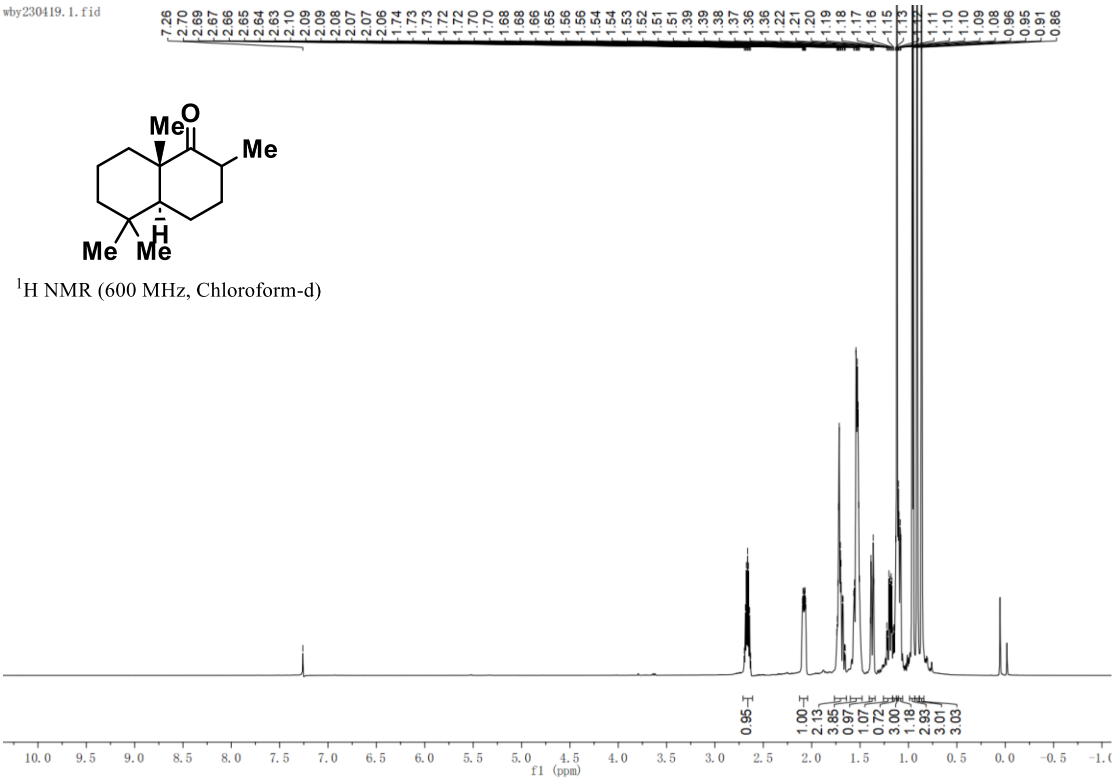

^{1}H NMR (600 MHz, Chloroform-d)

Compound 3 ^{13}C NMR

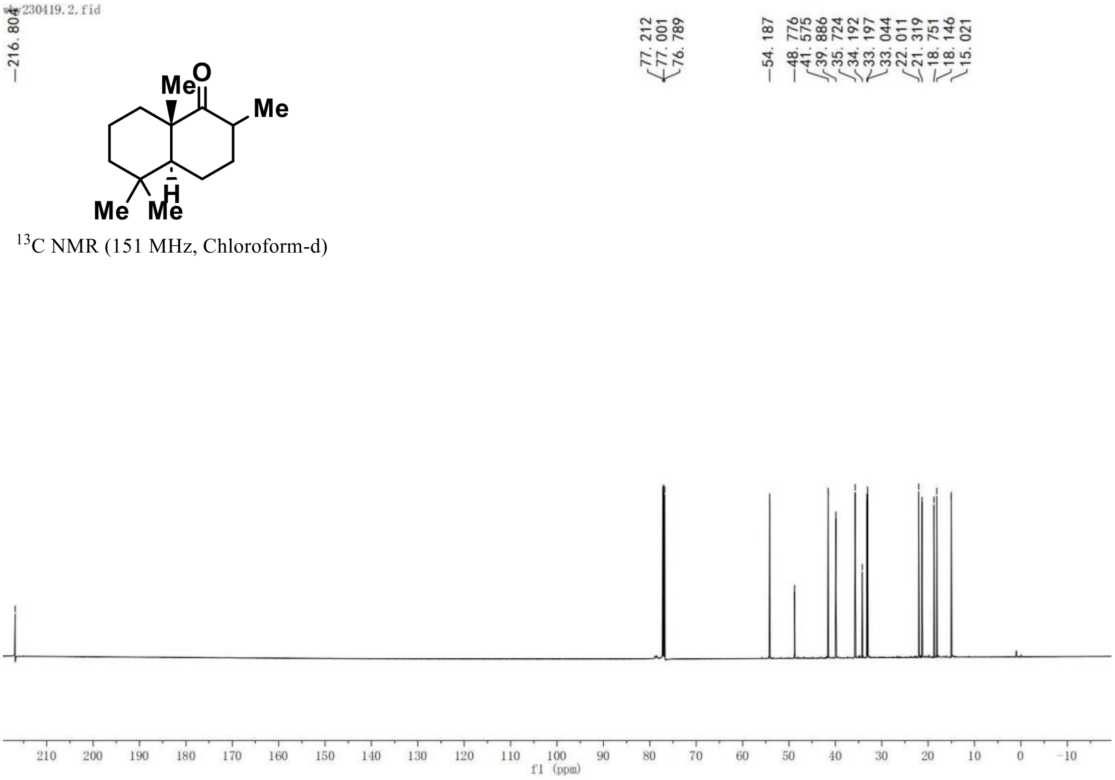

^{13}C NMR (151 MHz, Chloroform-d)

Compound 4 ^{1}H NMR

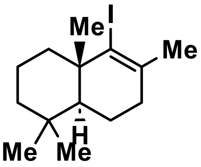

^{1}H NMR (600 MHz, Chloroform-d)

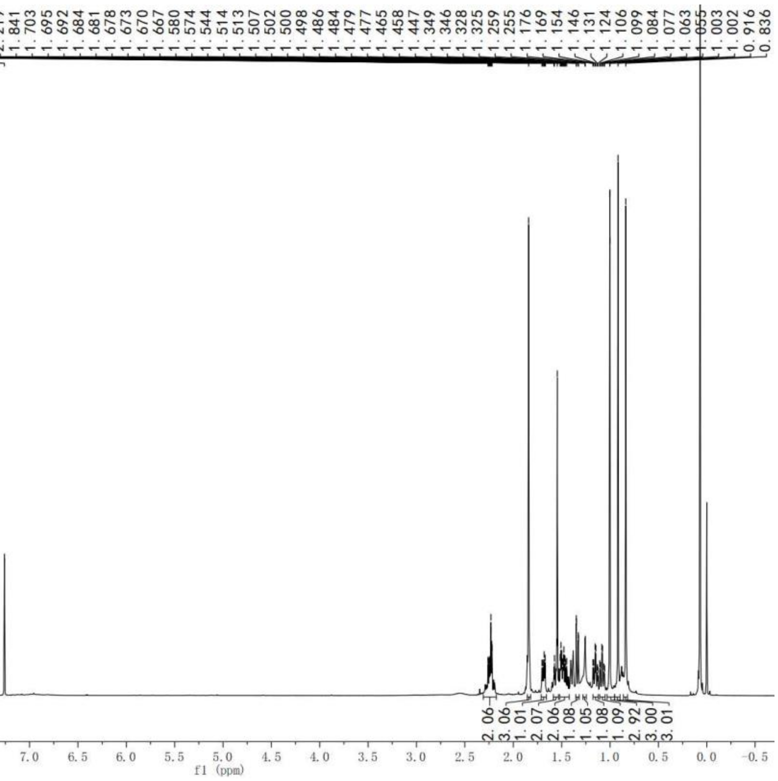

Compound 4 ^{13}C NMR

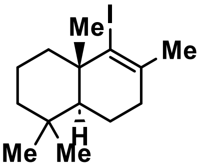

^{13}C NMR (151 MHz, Chloroform-d)

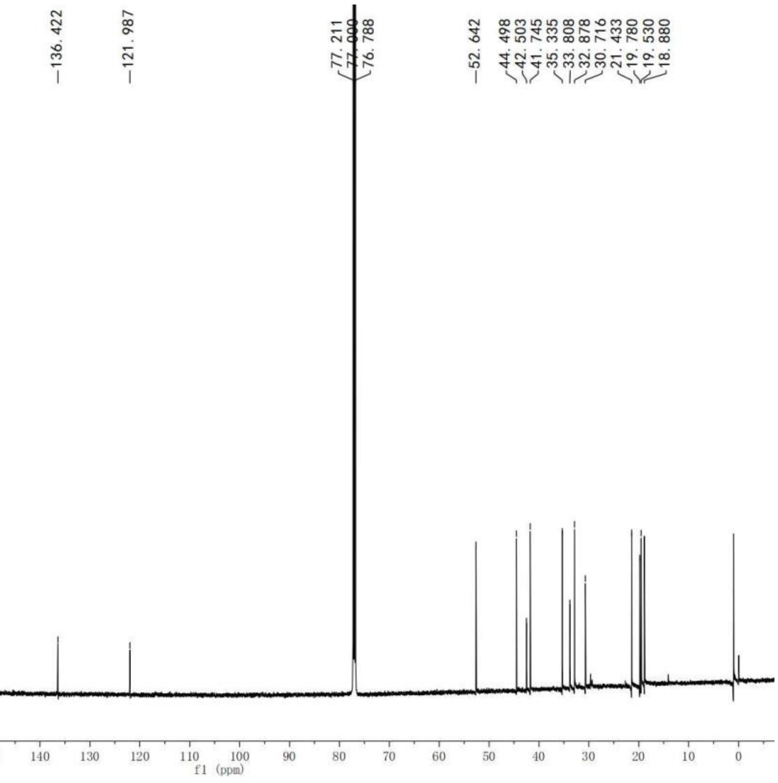

Compound 5 ^{1}H NMR

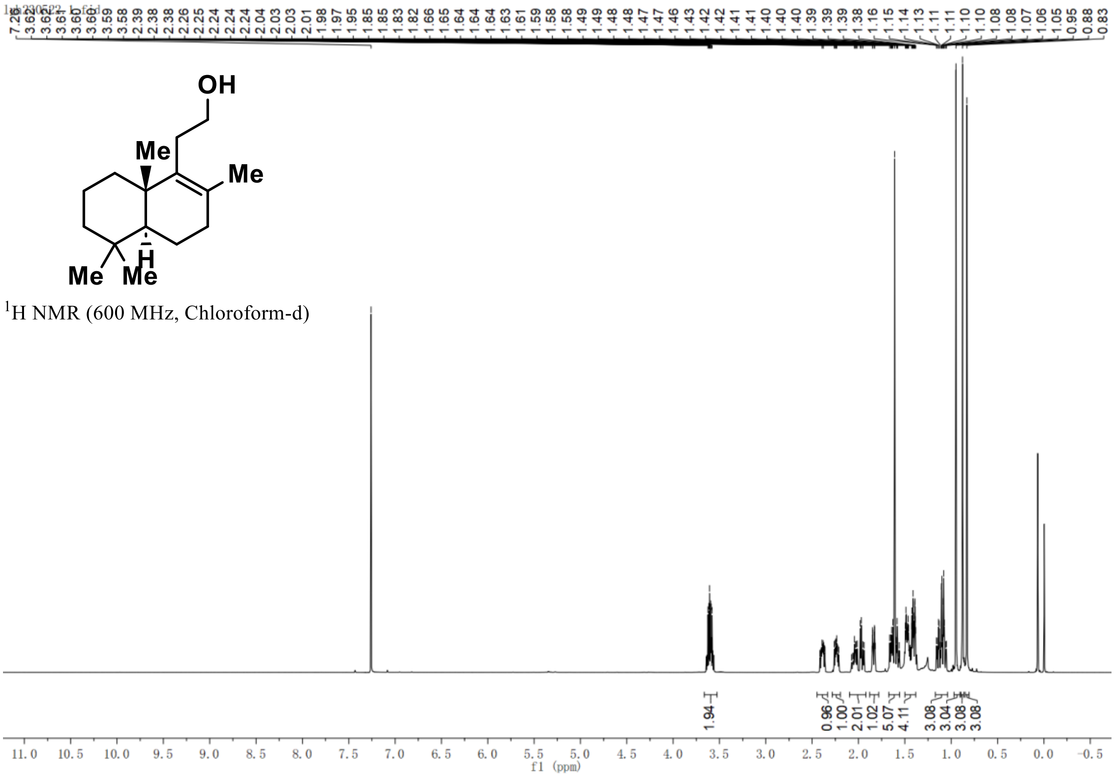

Compound 5 ^{13}C NMR

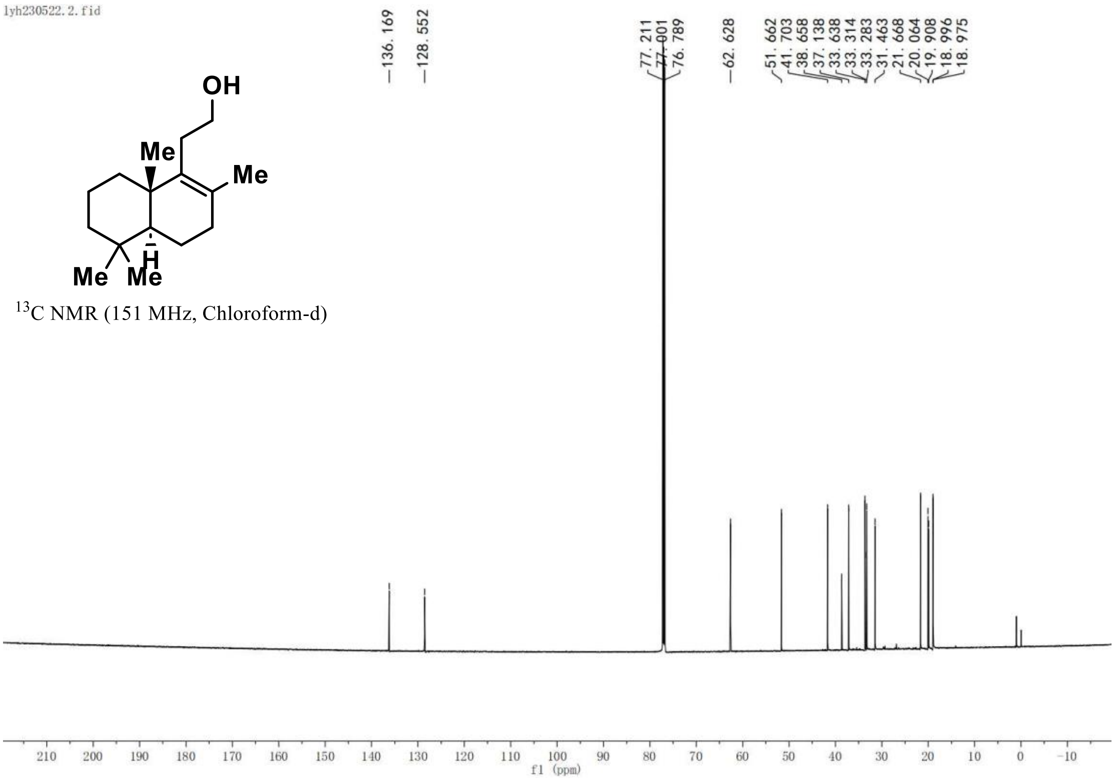

Compound (-)-ambrox ^{1}H NMR

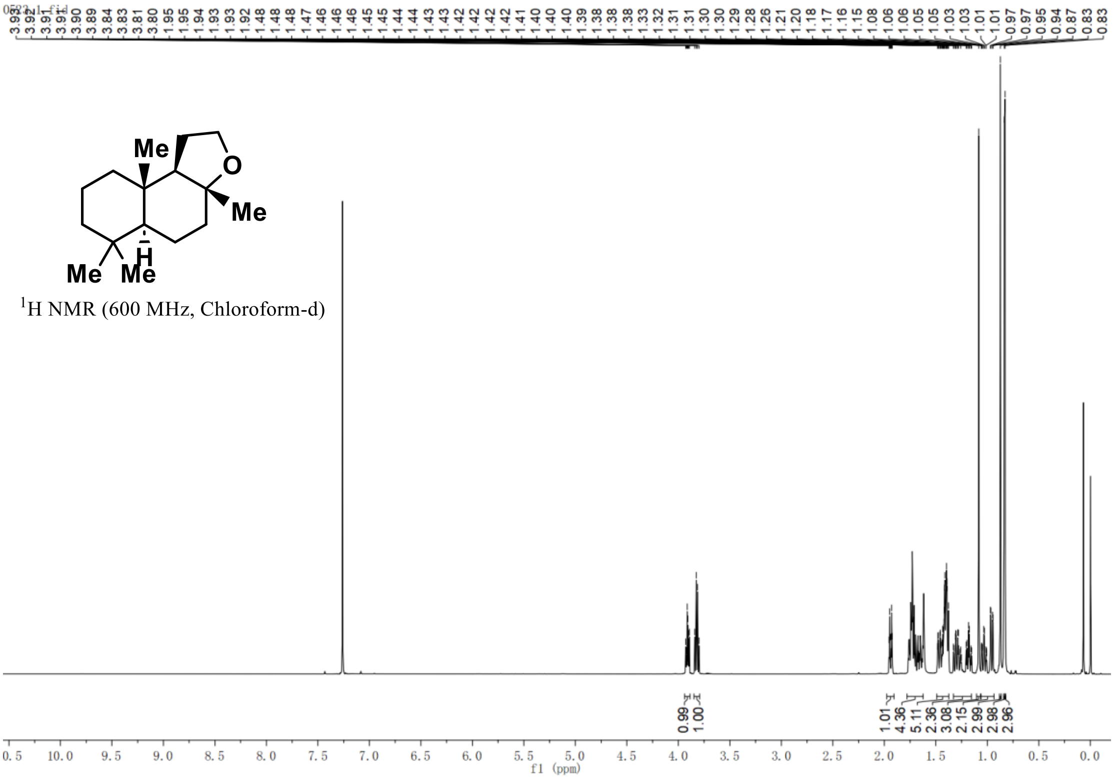

^{1}H NMR (600 MHz, Chloroform-d)

Compound (-)-ambrox ^{13}C NMR

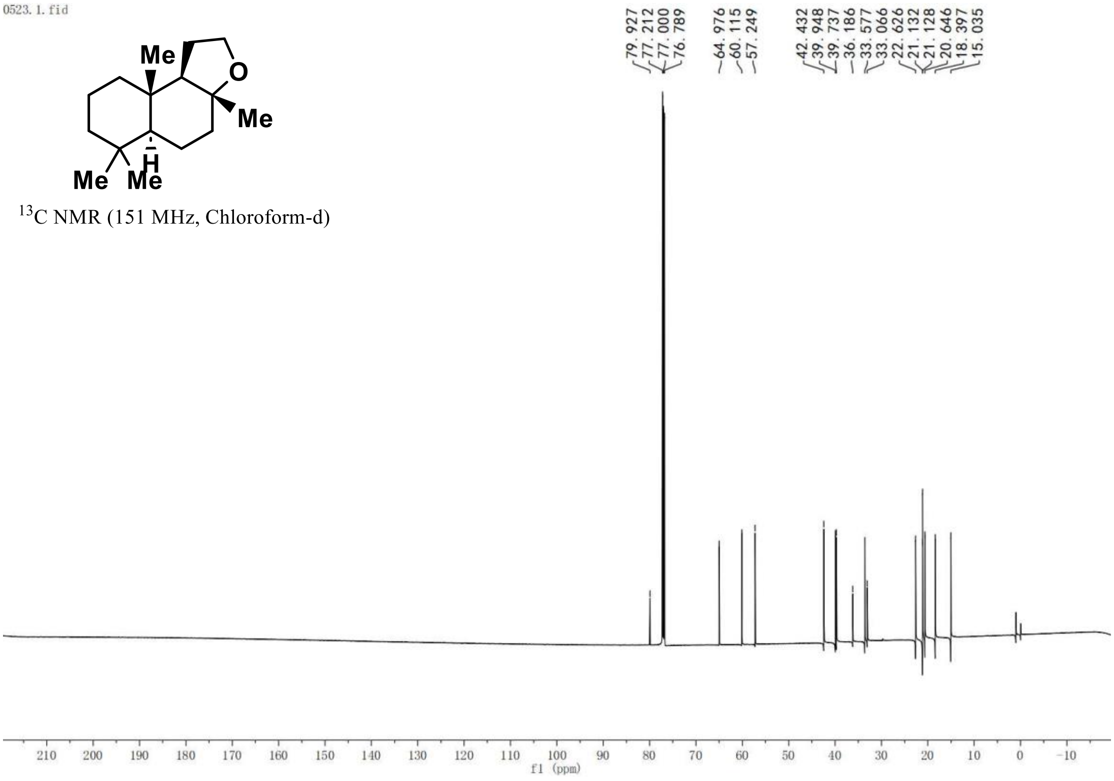

^{13}C NMR (151 MHz, Chloroform-d)

Comparison of the 1H NMR data for (-)-ambrox with the reported data

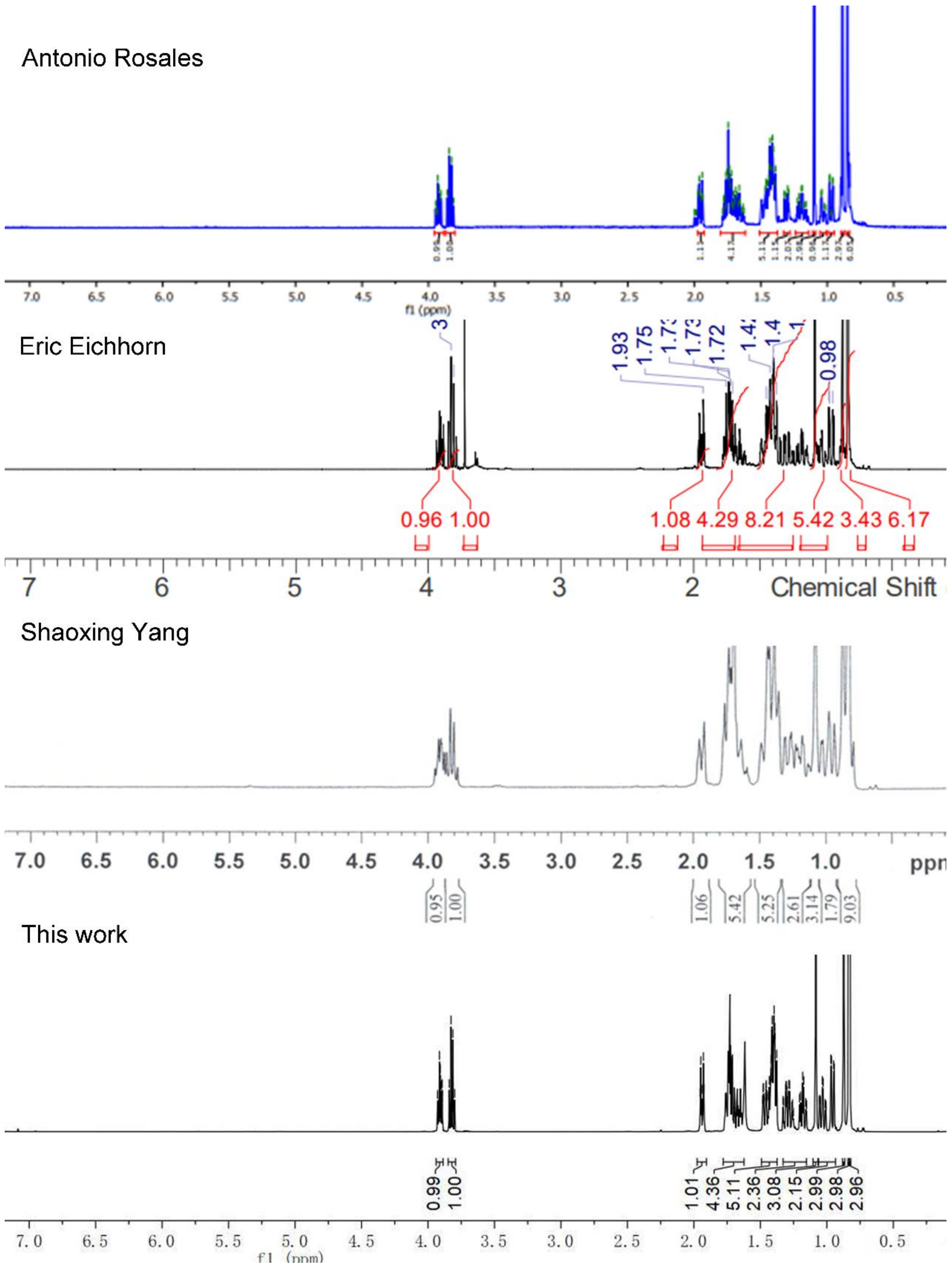

Synthetic (600 MHz)	Reported (400 MHz)
3.91 (td, J = 8.5, 3.8 Hz, 1H)	3.94 - 3.88 (m, 1H)
3.82 (q, *J* = 8.2 Hz, 1H)	3.82 (q, *J* = 8.2 Hz, 1H)
1.94 (dt, *J* = 11.6, 3.2 Hz, 1H)	1.94 (dt, *J* = 11.5, 3.0 Hz, 1H)
1.78 – 1.62 (m, 4H)	1.77 - 1.66 (m, 4H)
1.49 – 1.37 (m, 5H)	1.49- 1.37 (m, 5H)
1.33 – 1.15 (m, 2H)	1.35 - 1.14 (m, 2H)
1.08 (s, 3H)	1.09 (s, 3H)
1.06 – 0.93 (m, 2H)	1.09 - 0.95 (m, 2H)
0.87 (s, 3H)	0.88 (s, 3H)
0.83 (s, 3H)	0.84 (s, 3H)
0.83 (s, 3H).	0.83 (s, 3H)

Comparison of the ^{13}C NMR data for (-)-ambrox with the reported data

Antonio Rosales

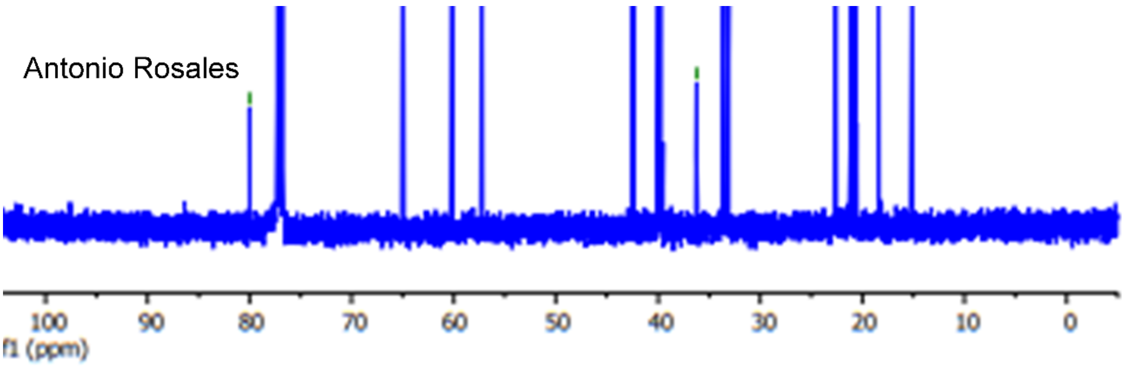

Eric Eichhorn

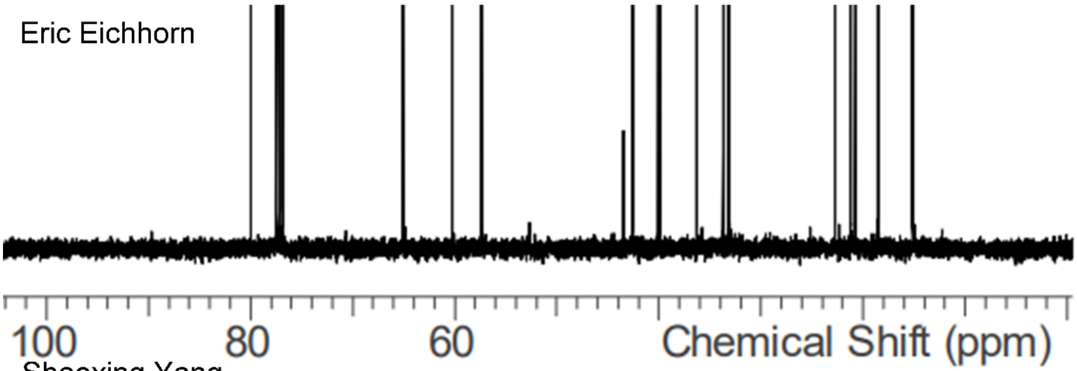

Shaoxing Yang

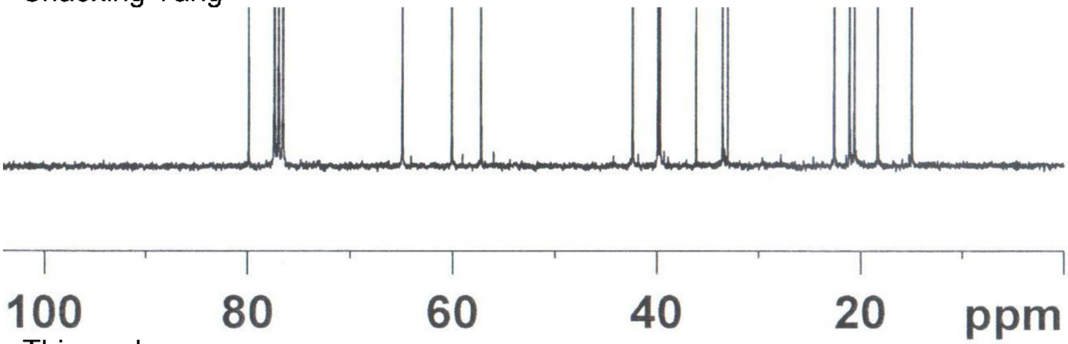

This work

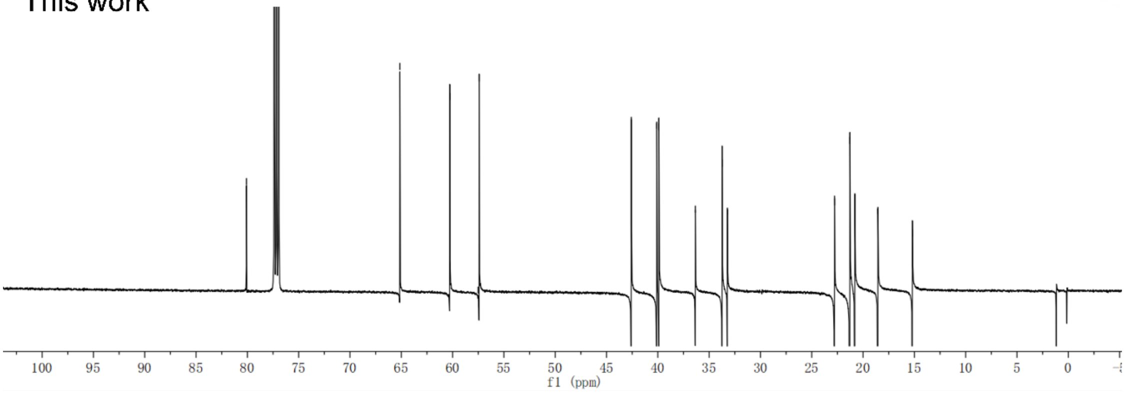

Synthetic (151 MHz)	Reported (100 MHz)
79.93	80.0
64.98	65.1
60.11	60.3
57.25	57.4
42.43	42.6
39.95	40.1
39.74	39.9
36.19	36.3
33.58	33.7
33.07	33.2
22.63	22.8
21.13	21.3
21.13	21.3
20.65	20.8
18.40	18.5
15.04	15.2

Comparison of the HRMS data for (-)-ambrox with the reported data

		Found	Requires
This work	$[M+H]^+$	237.2213	237.2218
Kenji Mori	M^+	236.2189	236.2140
Anja. A	M^+	236.2144	236.2140
Marjon G	M^+	236.2143	236.2140
